# Functionalized vertical GaN micro pillar arrays with high signal-to-background ratio for detection and analysis of proteins secreted from breast tumor cells

**DOI:** 10.1038/s41598-017-14884-x

**Published:** 2017-11-02

**Authors:** Mun-Ki Choi, Gil-Sung Kim, Jin-Tak Jeong, Jung-Taek Lim, Won-Yong Lee, Ahmad Umar, Sang-Kwon Lee

**Affiliations:** 1 Department of Physics, Chung-Ang University, Seoul, 06974 Republic of Korea; 2 Department of Chemistry, College of Science and Arts, and Promising Centre for Sensors and Electronic Devices (PCSED), Najran University, Najran, 11001 Saudi Arabia

## Abstract

The detection of cancer biomarkers has recently attracted significant attention as a means of determining the correct course of treatment with targeted therapeutics. However, because the concentration of these biomarkers in blood is usually relatively low, highly sensitive biosensors for fluorescence imaging and precise detection are needed. In this study, we have successfully developed vertical GaN micropillar (MP) based biosensors for fluorescence sensing and quantitative measurement of CA15-3 antigens. The highly ordered vertical GaN MP arrays result in the successful immobilization of CA15-3 antigens on each feature of the arrays, thereby allowing the detection of an individual fluorescence signal from the top surface of the arrays owing to the high regularity of fluorophore-tagged MP spots and relatively low background signal. Therefore, our fluorescence-labeled and CA15-3 functionalized vertical GaN-MP-based biosensor is suitable for the selective quantitative analysis of secreted CA15-3 antigens from MCF-7 cell lines, and helps in the early diagnosis and prognosis of serious diseases as well as the monitoring of the therapeutic response of breast cancer patients.

## Introduction

Encouraged by the recent progress in micro- and nano-fabrication, low-dimensional (e.g., 1D, 2D, and 3D) biosensors have been developed and widely used in numerous applications, including cancer cell imaging^1–4^ as well as biological detection and sensing^5–18^. Among these valuable applications, the detection of cancer biomarkers for determining the correct course of treatment with targeted therapeutics has also attracted significant attention^19–23^. Particularly, fluorescence-based biosensors allow the rapid and sensitive detection of various biomolecules, which is particularly helpful in the early diagnosis and prognosis of serious diseases and the monitoring of the therapeutic response of patients^3–7,24,25^. In this regard, the development of highly efficient biosensors is essential for the fluorescence imaging and precise detection of these biomolecules, which have a relatively low concentration in blood. For instance, typical circulating tumor markers are great importance for monitoring therapy in metastatic colorectal (carcinoembryonic antigen, CEA), prostate (prostate specific antigen, PSA), ovarian (carbohydrate antigen, CA125), breast (CA15-3 and CA 27.29), and pancreatic (CA 19-9) cancer patients^26–29^. Recently, many research groups have intensively explored the use of 2D materials to recognize target biomolecules with relatively low concentration. For instance, Xing and colleagues^11^ reported that a graphene-based optical sensor could enable the highly accurate label-free and live-cell detection of a small quantity of cancer cells. Yoon *et al*. also demonstrated an effective approach to isolating circulating tumor cells (CTCs) from blood samples of pancreatic, breast, and lung cancer patients by using functionalized graphene oxide on a patterned gold surface^12^. Recently, Lee and co-workers^15^ employed a MoS_2_ biosensor to electrically detect PSA with a highly sensitive and label-free method. These reports suggested that graphene-based materials and transition metal dichalcogenides are promising candidates for fabricating ultra-sensitive biosensors owing to their excellent conductivity^15,30^, high affinity to biomolecules^31–34^, and simple fabrication process of large-scale circuits. Moreover, 3D-based nanomaterials, including silicon nanowires^35,36^, quartz nanowires^1^, and TiO_2_ nanofibres^37^, have been used to capture CTCs, obtaining an excellent separation efficiency of >93% owing to an enhanced contact probability with biomolecules achieved by their high aspect ratios and contact areas.

In particular, the following tumor markers are associated with breast cancer: CEA, BRCA1, BRCA2, CA 15-3, and CA27.29^38,39^. Among these biomarkers, the CA15-3 antigen is an important and sensitive biomarker for the evaluation and monitoring of patients. However, because the concentration of the CA 15-3 biomarker in blood is usually relatively low, a highly sensitive biosensor is needed for fluorescence imaging and precise detection. To address this issue, we developed vertical GaN micropillar (MP) array-based biosensors for fluorescence sensing and quantitative measurement of the concentration of CA 15-3 antigens. Recently, geometry- and position-controlled GaN MPs have received significant attention as a promising template in a wide range of biological applications owing to their excellent transparency as well as mechanical and chemical stability^40–42^.

In this work, we grew vertical GaN MPs on the surface of hole-patterned GaN arrays with a metalorganic chemical vapor deposition (MOCVD) system using trimethylgallium (TMG) and high-purity NH_3_ gas^40^, and sub-cultured the MCF-7 cell line, that is known to secrete CA 15-3 and other proteins during cell growth^41,43^. Additionally, to detect breast tumor markers by an antibody-antigen interaction, we performed fluorescence sensing and quantitative measurements of secreted CA15-3 from MCF-7 cells using biotinylated CA15-3 antibody (CA15-3-Ab) functionalized streptavidin (STR)-GaN MP arrays. Based on our results, the highly ordered 3D structure of the GaN MP arrays resulted in a large surface area for immobilizing CA15-3 antigens on each feature of the arrays, which in turn led to enhanced fluorescence signals. Furthermore, the fluorescence-labeled antigen-functionalized MP arrays allowed the measurement of an individual fluorescence intensity from the top surface of the arrays owing to the regularity of the fluorophore-tagged MPs and relatively low background signal.

## Results and Discussion

### Growth of GaN MP Arrays

**Figure 1:**
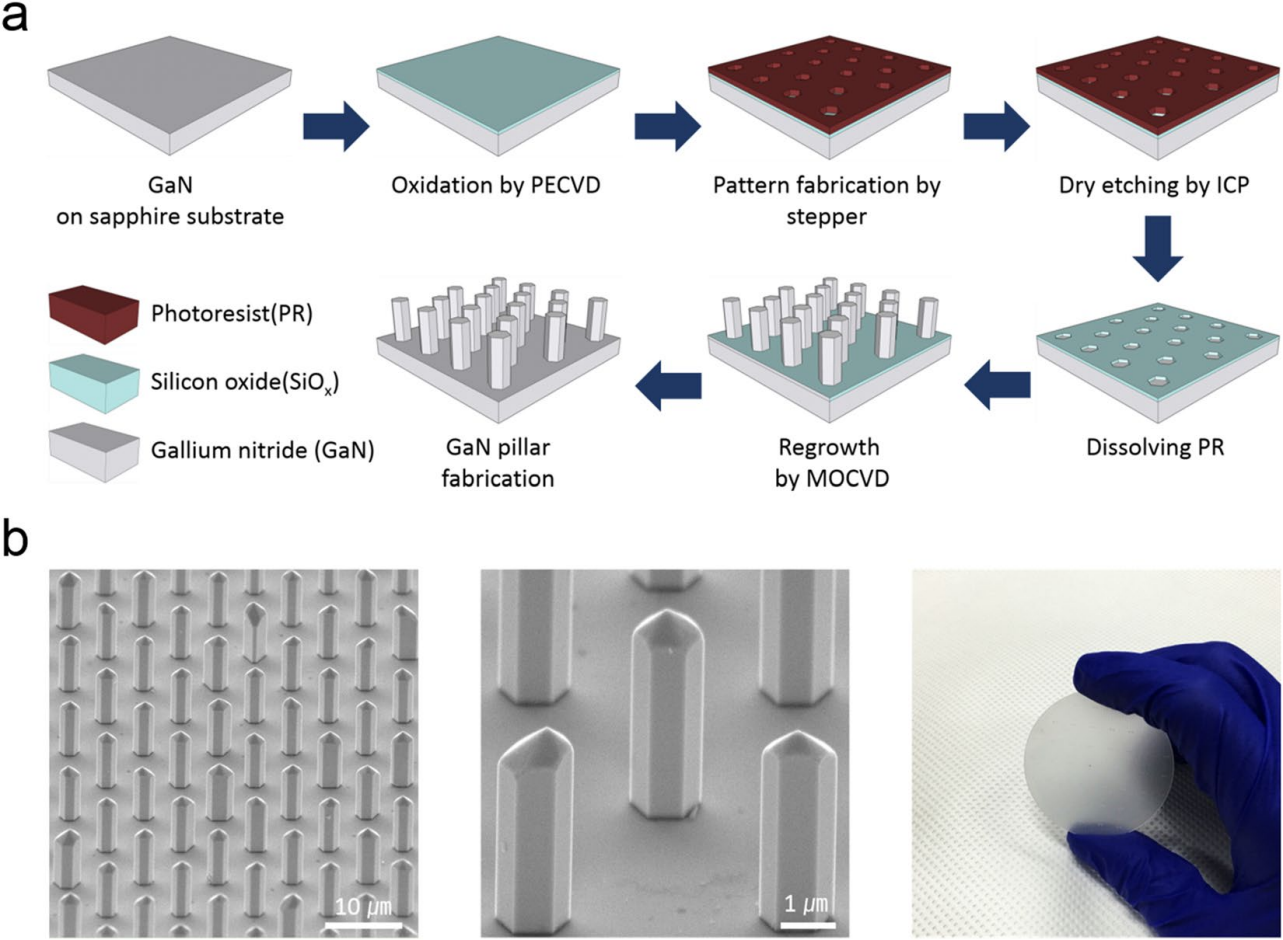
Growth of GaN MP arrays. (**a**) Schematic diagram of the GaN MP fabrication process: (1) growth of high-quality GaN films (thickness ~20 nm) on *c*-plane sapphire substrates with the MOCVD system; (2) deposition of thin SiO_2_ layer (thickness ~ 30 nm) with the PECVD system; (3) formation of photoresist hole patterns on the surface of the SiO_2_ film using a stepper lithography system; (4) anisotropic etching of the exposed SiO_2_ using an ICP-RIE; (5) chemical dissolution of the photoresist in acetone; (6) regrowth of GaN MP arrays on the surface of the hole-patterned GaN arrays with the MOCVD system; and (7) removal of remnant SiO_2_ film in a diluted HF solution. (**b**) SEM and optical images of as-grown GaN MP arrays on a 2-inch sapphire substrate after 300 cycles.

To facilitate fluorescence sensing and quantitative measurement of the CA15-3 antigen, a highly ordered 3D structure of GaN MP arrays was grown on the surface of hole-patterned GaN arrays with an MOCVD system using TMG as a Ga source and high-purity ammonia (NH_3_) as a N source. Figure 1(a) shows a series of process for the preparation of GaN MPs arrays. The detailed fabrication processes were described in the Materials and Methods and in previous reports^40–42^. Figure 1(b) shows scanning electron microscopy (SEM) and optical images of as-grown GaN MP arrays on a 2-inch sapphire substrate. The average diameter, length, and density of the MPs were determined to be ~1.2 μm, ~3.5 μm, and 230,000 MPs/mm^2^, respectively, corresponding to a distance of ~3.2 μm between MPs. According to the previous results reported by Martinez and co-workers^5^, the nanostructures must be at least ~2 μm high and have more than 400 nm spacing to achieve the desirable fluorescence imaging. Therefore, our GaN MP arrays are suitable for facilitating fluorescence sensing and quantitative analysis of the CA15-3 antigens secreted from MCF-7 cells.

### Surface Functionalization of GaN MP Arrays for Biomarker Detection

**Figure 2:**
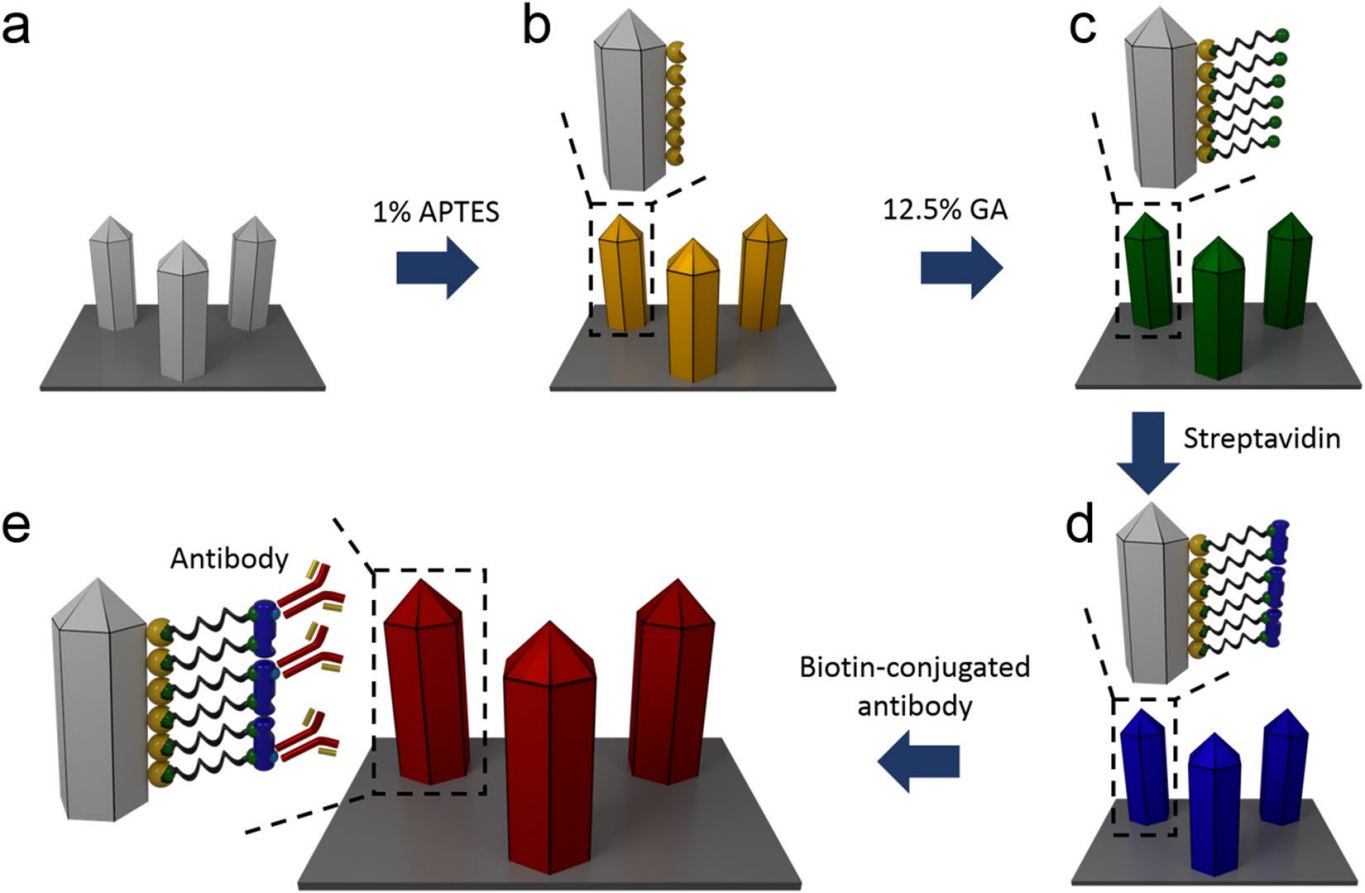
Surface functionalization of as-grown GaN MP arrays. (**a**) O_2_ plasma treatment of GaN MP arrays; (**b**) formation of amine groups on the surface of the GaN MPs with a 1% APTES solution; (**c**) immobilization of the aldehyde group on APTES-coated GaN MPs; (**d**) immobilization of STR to GA; and (**e**) functionalization of CA 15-3-Ab on the STR-conjugated GaN MPs.

To fabricate a GaN MP array-based biosensor for biomarker detection, the surface of the MP arrays was chemically functionalized through a series of processes, as shown in Fig. 2. In particular, the surface of the GaN MPs was treated with O_2_ plasma for 20 s to confer the hydroxyl groups on the surface and then was modified with 1% (v/v) APTES in ethanol and at room temperature for 30 min using a 3D-rocker (100 rpm), resulting in the attachment of the amine group to the surface, as shown in Fig. 2(a,b). Our previous study suggested that this treatment plays an important role in streptavidin (STR, Sigma-Aldrich, USA) immobilization on topographical substrates because the hydroxyl groups provide the nanostructured arrays with enhanced conjugation with 3-APTES (Sigma-Aldrich, USA)^33,44^. After rinsing with an ethanol solution for 10 min, the GaN MPs were placed on a hot plate at 120 °C for 10 min and reacted with 12.5% (v/v) GA (Sigma-Aldrich, USA) in distilled water on the 3D-rocker for 4 h, leading to the immobilization of the aldehyde group on the APTES-coated GaN MPs (Fig. 2(c)). Subsequently, the STR was immobilized to GA by incubating the GaN MPs with a 20 µg/mL STR solution in PBS in an incubator (37 °C, 5% CO_2_) overnight. Finally, the STR-conjugated GaN MPs (STR-GaN MPs, Fig. 2(d)) were chemically functionalized with the CA15-3-Ab (×500 dilution) in the incubator (4 °C, 5% CO_2_) for 24 h and rinsed with PBS solution, resulting in CA15-3-Ab-functionalized STR-GaN MPs (Fig. 2(e)).

**Figure 3:**
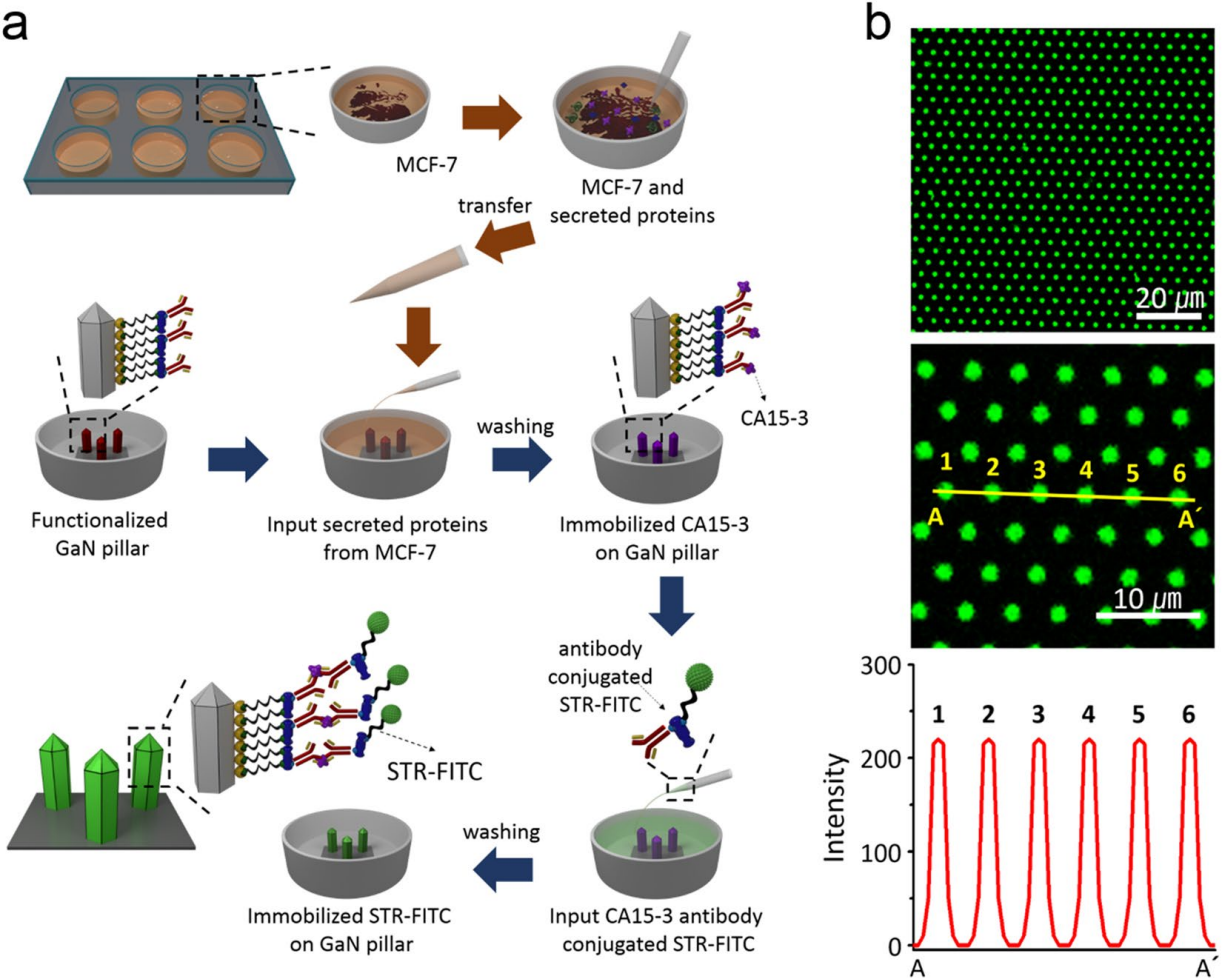
CA15-3 detection on the functionalized GaN MP arrays. (**a**) Schematic representation of the series of processes used for fluorescence detection and quantitative analysis of the secreted CA15-3 antigen: (1) extraction of proteins from MCF-7 suspension, (2) immobilization of CA 15-3 antigen on the CA15-3-Ab-functionalized STR-GaN MP arrays, and (3) staining of the immobilized CA15-3 antigens with FITC- STR. (**b**) Representative top-view images of the fluorescence-activated GaN MPs and line profile for six MPs along the yellow line in the magnified fluorescence image (A-A′).

To detect breast tumor markers by an antibody-antigen interaction, the CA15-3-Ab-functionalized STR-GaN MPs were mainly used in this study. Figure 3(a) shows the schematic representation of a series of processes for fluorescence sensing and quantitative measurement of CA15-3 secreted from MCF-7 cells. In brief, proteins extracted from an MCF-7 suspension were reacted with the CA15-3-Ab-functionalized STR-GaN MPs at 4 °C for 20 min. Then, the immobilized CA15-3 antigens on the functionalized GaN MPs were stained with a CA15-3-Ab-conjugated fluorescein isothiocyanate-labeled STR (STR-FITC) solution in the incubator for 24 h and fixed with 4% paraformaldehyde solution (PFA, Santa Cruz Biotechnology Inc., USA). Finally, the STR-FITC-immobilized GaN MP arrays were imaged with a confocal microscopy system (LSM 700, Zeiss, USA). Figure 3(b) shows representative top-view images of the fluorescence-activated GaN MPs and the corresponding line profile through the six MPs along the yellow line marked in the magnified fluorescence image (A-A′). These results demonstrate that the CA15-3-Ab-functionalized STR-GaN MPs homogenously immobilized CA15-3 antigens on each feature of the arrays, which led to uniform fluorescence brightness in a large given area. Furthermore, the fluorescence-labeled antigen-functionalized GaN MP arrays allowed us the measurement of an individual fluorescence intensity from the top surface of the arrays owing to the high regularity of fluorophore-tagged MPs and the relatively low background signal.

### Quantitative Analysis of CA 15-3 Antigen using GaN-MP-based Biosensor

**Figure 4:**
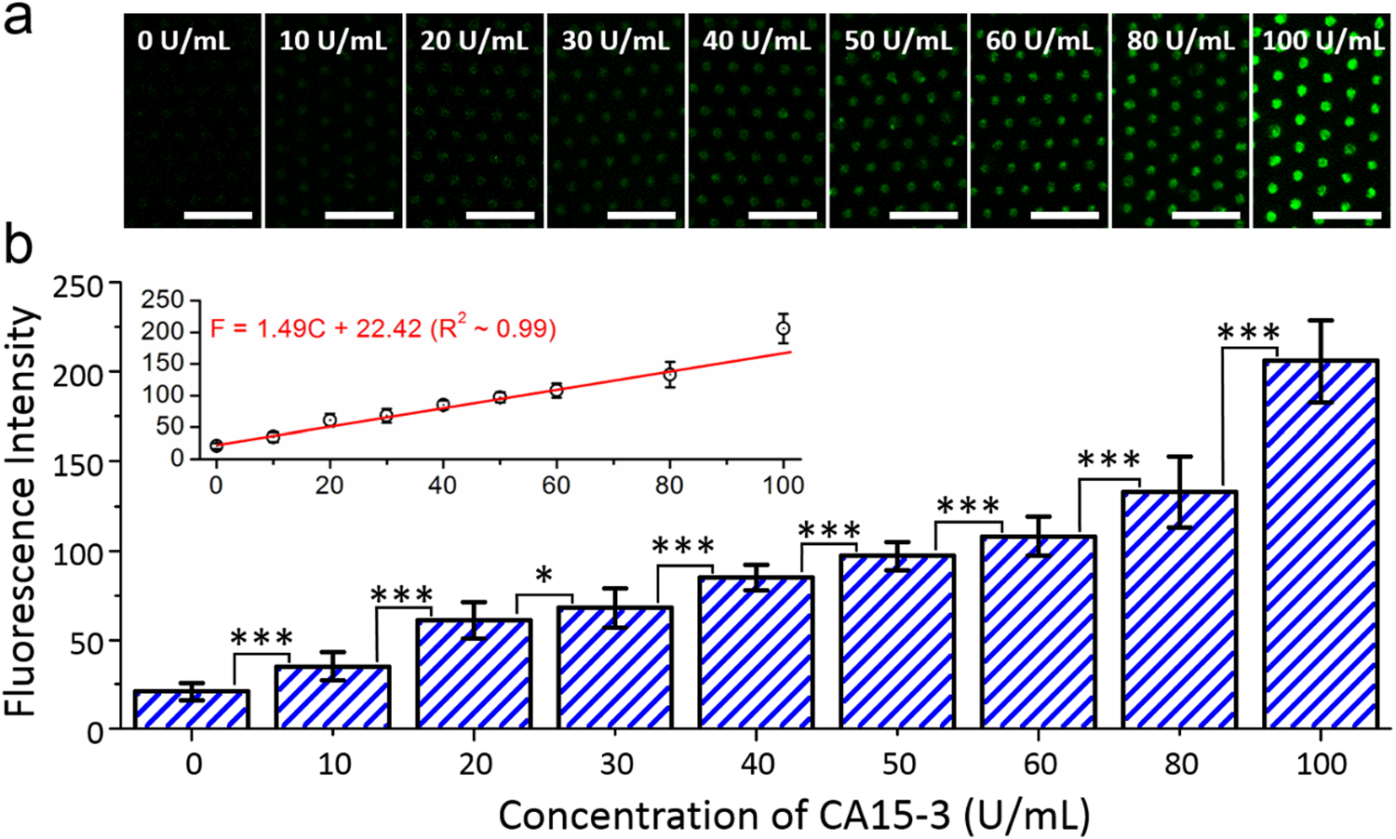
Fluorescence detection of the CA 15-3 antigen using GaN-MP-based biosensors. (**a**) Fluorescence images and (**b**) intensities dependent on the concentration of the CA15-3 antigen (up to 100 U/mL) bound on the functionalized GaN MPs (n = 3, 63 GaN MPs). The statistical significance of the fluorescence intensity dependent on the concentration of CA 15-3 antigen was assessed using student unpaired t-test. *P < 0.05, **P < 0.01, ***P < 0.001. The inset of (**b**) shows the fluorescence intensity as a function of the concentration of the CA15-3 antigen.

To quantify the fluorescence intensity, which is dependent on the concentration of the CA15-3 antigen, we prepared various solutions containing 0, 10, 20, 30, 40, 50, 60, 80, and 100 U/mL CA15-3 antigens. After reacting these solutions with the CA15-3-Ab-functionalized STR-GaN MPs at 4 °C for 20 min, all of the immobilized CA15-3 antigens on the GaN MPs were stained with the CA15-3-Ab-conjugated STR-FITC solution in the incubator for 24 h and fixed with a 4% PFA (Santa Cruz Biotechnology Inc., USA) solution to indirectly detect fluorescence images and intensities. As shown in Fig. 4(a), the fluorescence images were detected from all of the CA15-3-immobilized GaN MPs. In the case of a 0 U/mL CA15-3 antigen solution, the fluorescence intensity was determined to be approximately 21 ± 5. This originated from the non-specific antigen-antibody binding and the high affinity between the biomolecule and the hydrophobic materials^32,45,46^, which, in turn, attached a small quantity of fluorescence material on the surface of the GaN MPs. By increasing the concentration of CA15-3 antigens to 80 U/mL, the fluorescence intensities gradually increased to 133 ± 20 (shown in Fig. 4(b) and Table 1), which was correlated with the increase of CA15-3-Ab-conjugated STR-FITCs on the CA15-3-immobilized GaN MPs. In the case of a 100 U/mL CA15-3 antigen solution, on the other hand, the fluorescence intensity was determined to be 206 ± 23 and significantly higher than measured values on the low concentration in the range 0–80 U/mL. Based on these results, the fluorescence intensity showed a good linear correlation (R^2^ ~ 0.99) with the concentration of the CA15-3 antigen in the range from 0 to 80 U/mL, which was similar to previous publication reported by Shadfan and co-workers^47^. They also demonstrated the linear relationship between the mean fluorescence intensity and the low concentration of biomarkers (i.e., CA 125, HE4, MMP-7, and CA 72-4). By plotting these data, the fluorescence intensity is defined by the following regression equation:

1 $${\rm{F}}=1.49{\rm{C}}+22.42$$

where C is the concentration of the CA15-3 antigen. Because the cut-off value for CA 15-3 antigen as a breast cancer marker is known to be ~30 U/mL^48^, our GaN-MP-based biosensor can potentially determine the concentration of CA 15-3 antigens in patients with breast cancer using this regression equation.

**Table 1:** Measured fluorescence intensity on the functionalized GaN-MP-based biosensor as a function of loaded CA 15-3 concentration (n = 3, 63 GaN MPs).

Loaded CA 15-3 concentration (U/mL)	Measured Fluorescence intensity
0	21 ± 5
10	35 ± 8
20	61 ± 10
30	68 ± 11
40	85 ± 7
50	97 ± 8
60	108 ± 11
80	133 ± 20
100	206 ± 23

### Selective Detection of CA 15-3 Antigen Secreted from MCF-7 cells

**Figure 5:**
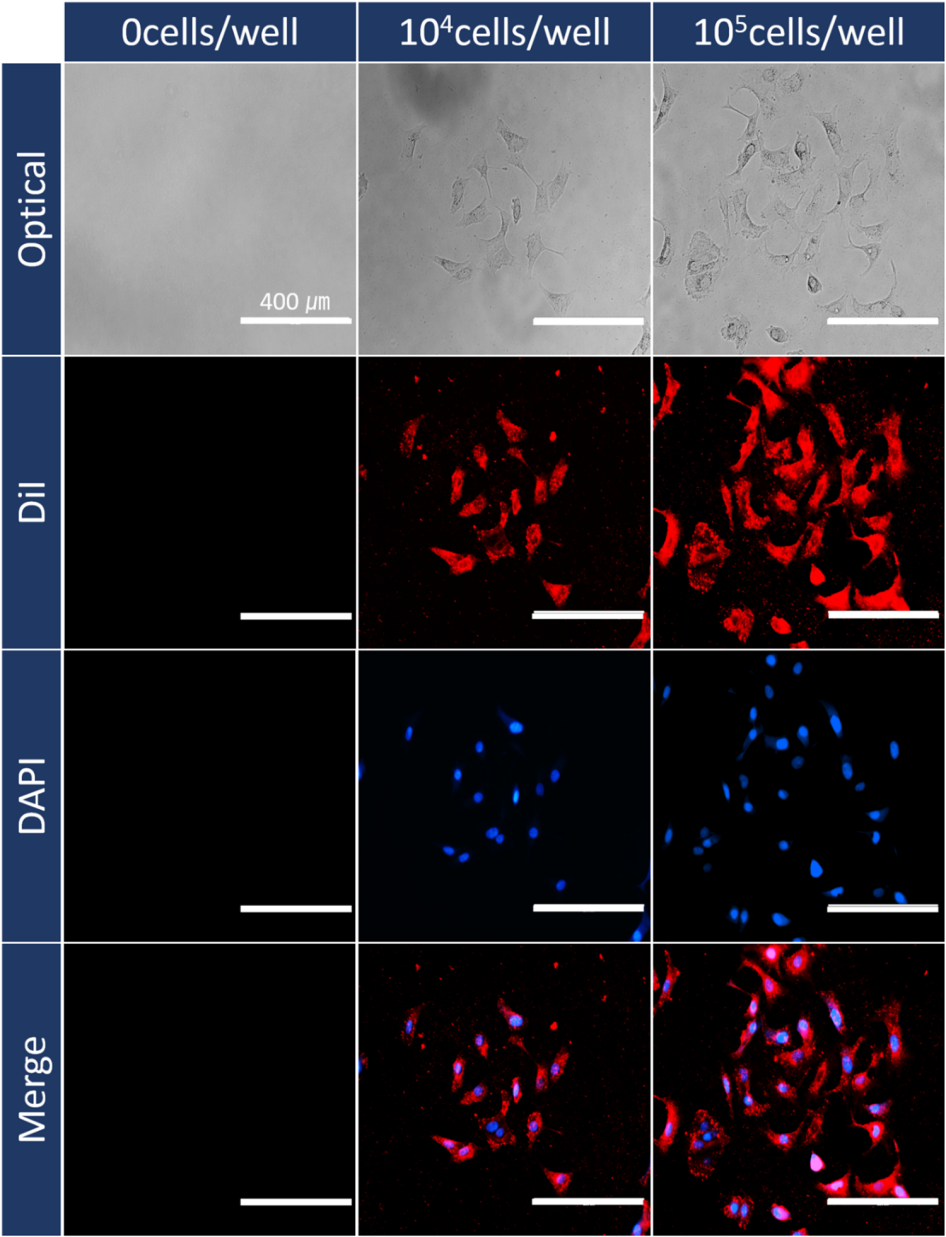
Optical and florescence images. Optical and overlapped fluorescence images of cultured MCF-7 cells with different cell populations (0, 10^4^, and 10^5^ cells/well). All the cells were stained with DiI (Vybrant cell-labeling solution; membrane staining; 565 nm; Thermo Fisher Scientific, USA) and DAPI (4′,6-diamidino-2-phenylindole; nuclear staining; 470 nm; Thermo Fisher Scientific, USA).

**Figure 6:**
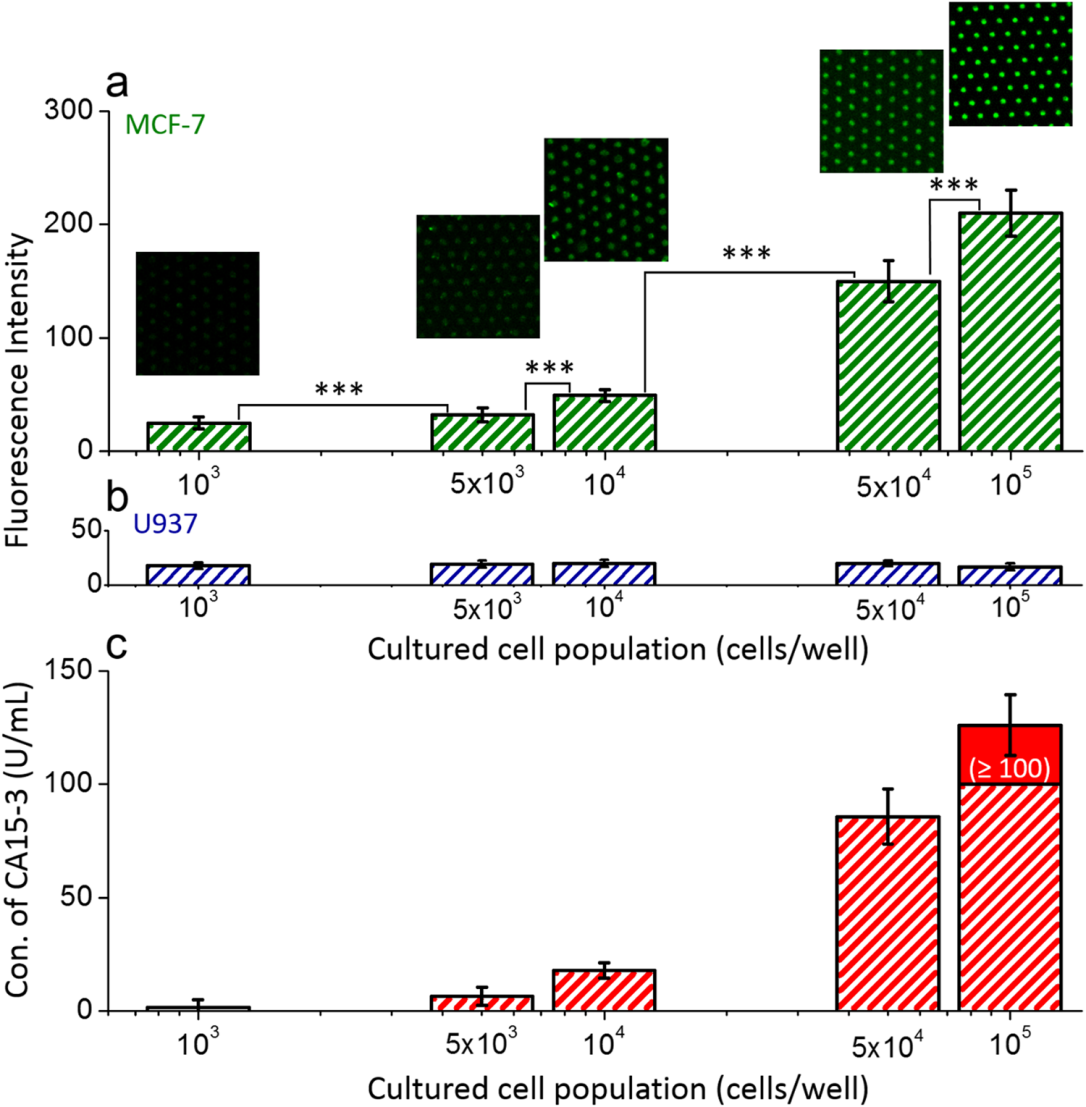
Selective detection of CA 15-3 antigen secreted from MCF-7 cells. (**a**) Fluorescence images and intensities of the surface of the GaN MP arrays; the immobilized FITC-labeled STR bound on the CA 15-3 antigens were extracted from a cultured suspension containing MCF-7 cell populations of up to ~10^5^ cells/well (n = 3, 63 MPs). The statistical significance of fluorescence intensity dependent on the population of MCF-7 cells was assessed using student unpaired t-test. *P < 0.05, **P < 0.01, ***P < 0.001. (**b**) Fluorescence intensity of the surface of the GaN MP arrays reacted with extracted proteins from the cultured U937 suspensions. (**c**) Calculated concentrations of secreted CA 15-3 antigens for cultured MCF-7 cell populations of up to 1 × 10^5^ cells/well.

To identify the selective detection and quantitative measurement of CA 15-3 antigens secreted from different cell populations, we sub-cultured the MCF-7 cells with the populations of ~1 × 10^3^, ~5 × 10^3^, ~1 × 10^4^, ~5 × 10^4^, and ~1 × 10^5^ cells/well during 76 h and confirmed the cell viability using optical and overlapped fluorescence images (Fig. 5), respectively. After reacting the extracted proteins from the cultured MCF-7 suspensions with the functionalized GaN-based biosensors and subsequent STR-FITC immobilization, the fluorescence images and intensities were measured to assess the concentration of CA15-3. As shown in Fig. 6(a), the fluorescence brightness and its intensity are gradually enhanced by increasing loaded cell populations. The intensities of the CA 15-3 antigens secreted from MCF-7 were determined to be 25 ± 5, 32 ± 6, 49 ± 5, 150 ± 18, and 210 ± 20, respectively, for the initially loaded MCF-7 cell populations of ~1 × 10^3^, ~5 × 10^3^, ~1 × 10^4^, ~5 × 10^4^, and ~1 × 10^5^ cells/well, as shown in Fig. 6(a) and Table 2. Based on the linear relationship between the fluorescence intensity and the concentration of CA15-3 antigens defined by equation (1), as shown in Fig. 6(c), the corresponding concentrations of the CA 15-3 antigens secreted from MCF-7 were determined to be 1.7 ± 3.4, 6.4 ± 4.0, 17.8 ± 3.4, and 85.6 ± 12.1 U/mL, respectively. On the other hand, the highest fluorescence intensity of 210 ± 20 was inevitably determined to be ≥100 U/mL because its intensity located above the linear range. Additionally, the functionalized GaN-based biosensors were reacted with the extracted proteins from the cultured U937 suspensions. Since the U937 cell line as a lung cancer marker does not secrete the CA 15-3 antigen during cell growth, we easily confirm the selective detection of the CA 15-3 antigen. As shown in Fig. 6(b), the fluorescence intensity was almost similar in all U937 suspensions, indicating that a small quantity of fluorescence material was mainly attached on the surface of the GaN MPs caused by non-specific antigen-antibody binding. Therefore, the fluorescence-labeled GaN-MP-based biosensor is suitable for the selective fluorescence sensing of secreted CA15-3 antigens from MCF-7 cells, and allowing the quantitative analysis of breast tumor markers as well as the monitoring of breast cancer patient therapy.

**Table 2:** CA 15-3 concentration calculated from the dependence of the measured fluorescence intensity on the cultured MCF-7 cell population (n = 3, 63 GaN MPs).

Cultured MCF-7 cell population (cells/well)	Measured fluorescence intensity	Calculated CA 15-3 concentration (U/mL)
1 × 10^3^	25 ± 5	1.7 ± 3.4
5 × 10^3^	32 ± 6	6.4 ± 4.0
1 × 10^4^	49 ± 5	17.8 ± 3.4
5 × 10^4^	150 ± 18	85.6 ± 12.1
1 × 10^5^	210 ± 20	126 ± 13.4 (≥100)

**Figure 7:**
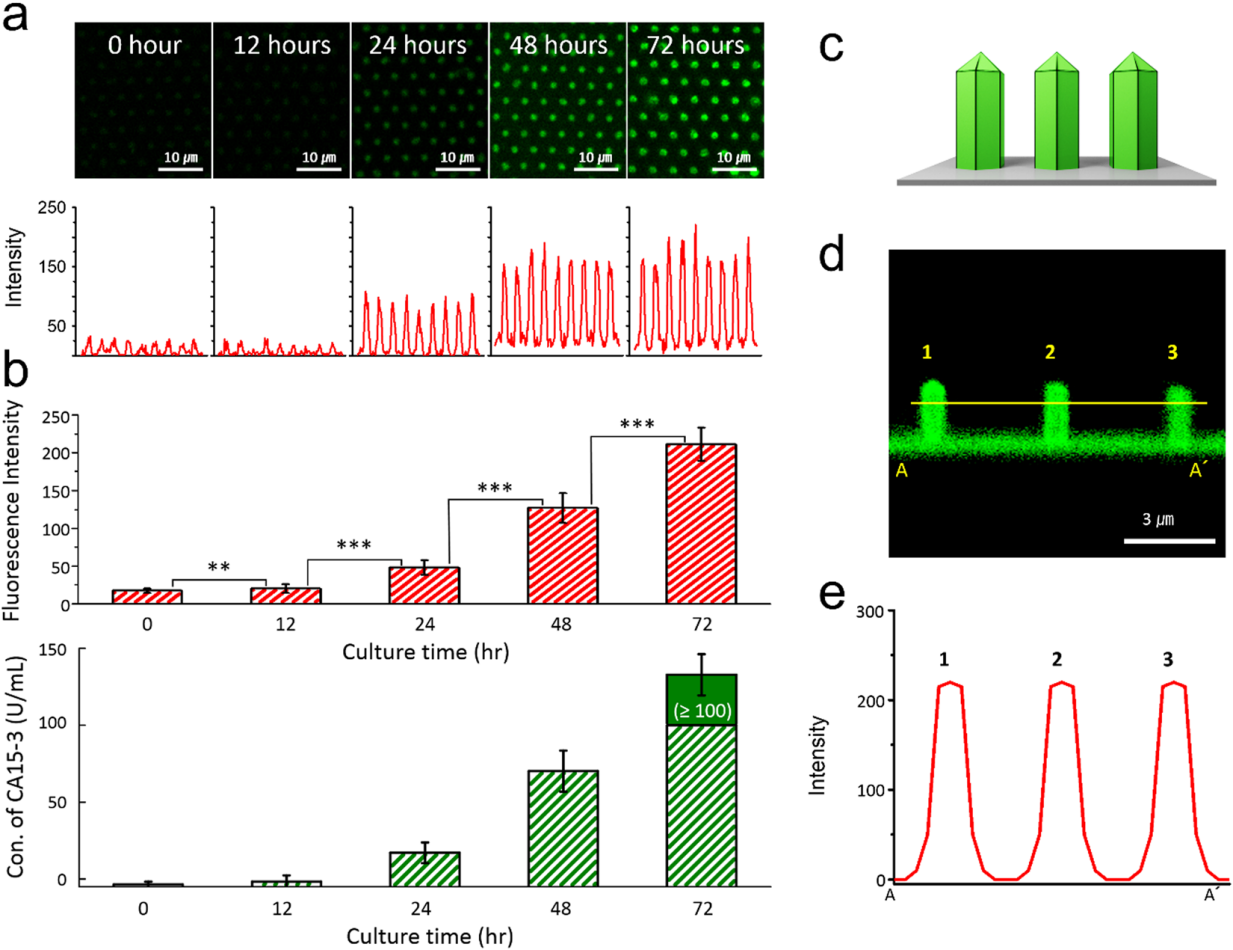
Quantitative analysis of the secreted CA 15-3 antigen as a function of culture time. (**a**) Fluorescence images and line profiles (through nine MPs) on the surface of the GaN MP arrays; the immobilized FITC-labeled STR bound on the CA 15-3 antigens were extracted from a suspension of MCF-7 cells (1 × 10^5^ cells/well) cultured for up to 72 h (n = 3, 21 GaN MPs). The statistical significance of the fluorescence intensity as a function of the culture time was assessed using student unpaired t-test. *P < 0.05, **P < 0.01, ***P < 0.001. (**b**) Fluorescence intensities and calculated concentrations of secreted CA 15-3 antigens as a function of culture time. (**c**) Cross-sectional illustration of three GaN MPs. (**d**) Fluorescence signal in the cross-sectional direction of the three illustrated MPs. (**e**) Line profile obtained below the tips of the GaN MPs along the yellow line (A-A′) marked in (**d**).

**Figure 8:**
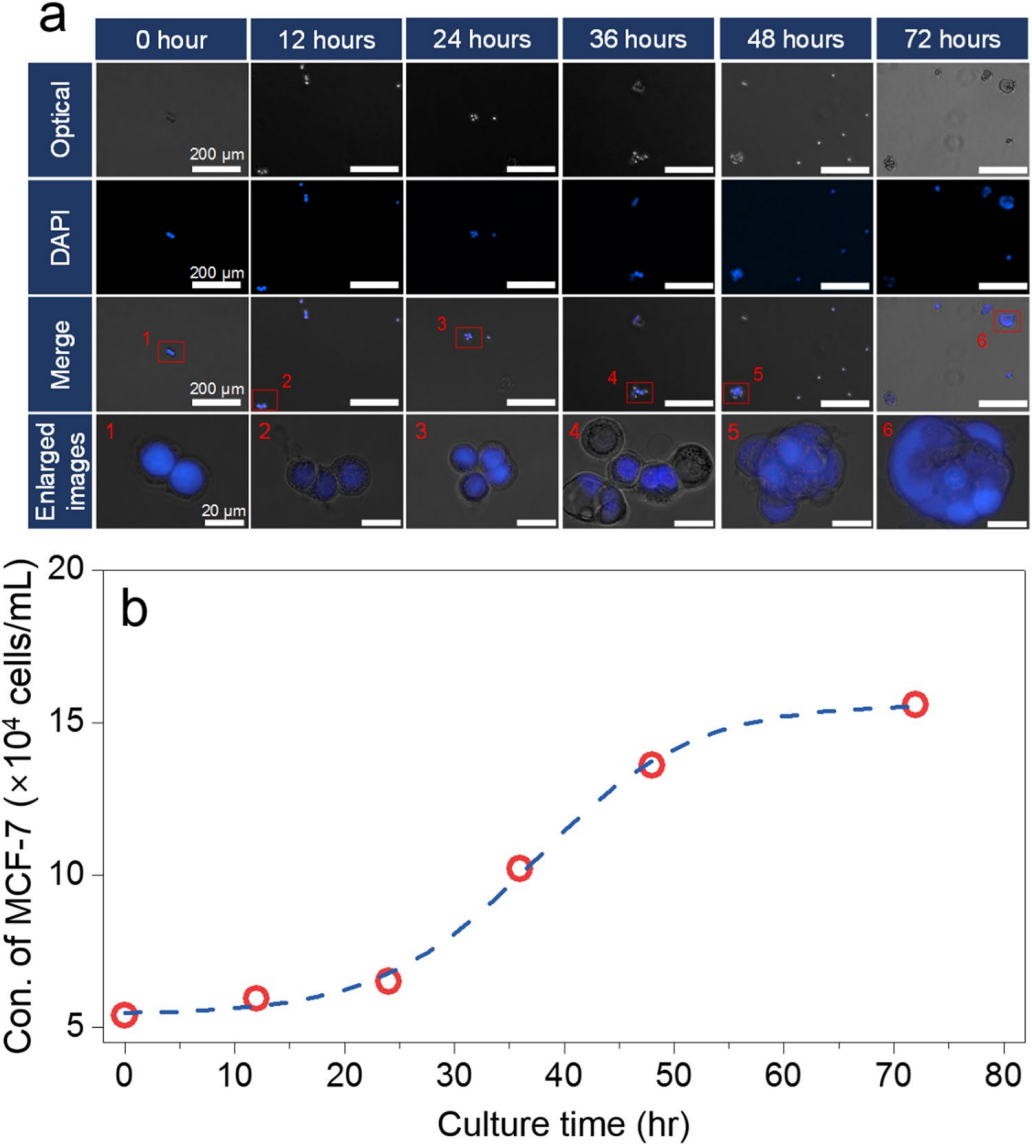
Overlapped fluorescence images and evaluated populations of MCF-7 cell dependent on culture time. (**a**) Optical and overlapped fluorescence images and (**b**) evaluated populations of cultured MCF-7 cells as a function of culture time (0, 12, 24, 36, 48, and 76 h). All the cells were stained with DAPI (4′,6-diamidino-2-phenylindole; nuclear staining; 470 nm; Thermo Fisher Scientific, USA).

**Figure 9:**
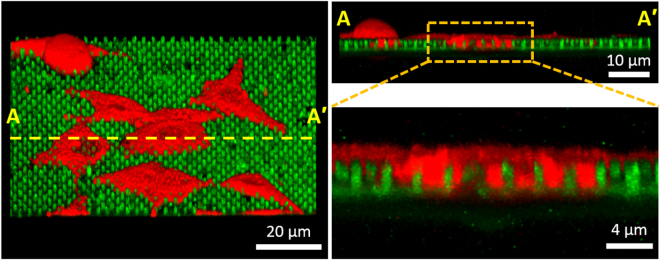
Confocal fluorescence image of MCF-7 cells on GaN MP arrays. (**a**) Surface and (**b**) cross-sectional confocal fluorescence images of living MCF-7 cells interfaced with a GaN MP array of ~3.5-μm-long and ~3.2-μm-spaced MPs. The GaN MP array were sterilized by immersing in ethanol for 30 min followed by exposure to UV for 1 h prior to seeding the MCF-7 cells. The GaN MPs and MCF-7 cells were stained with STR-FITC and DiI, respectively. The cross-sectional fluorescence image was obtained by confocal z-stacks along the marked yellow line (A-A′).

To further quantify the concentration of CA15-3 antigens as a function of culture time (up to 3 days) by using MCF-7 cell solutions containing ~1 × 10^5^ cells/well, we measured the fluorescence images and intensity on the surface of the GaN MPs. As shown in Fig. 7(a), the fluorescence brightness and line-scanned intensity along nine GaN MPs are gradually enhanced for increasing culture time. Based on these results, the CA 15-3 antigens were continually secreted from MCF-7 cells during cell growth, and the fluorescence intensities along the nine MPs were measured to be 16.5 ± 2.5, 20.2 ± 5.7, 48.1 ± 10.1, 127.4 ± 20.0, and 210.5 ± 21.6. Furthermore, as shown in Fig. 7(b) and Table 3, the concentration of secreted CA 15-3 antigens was determined to be −3.6 ± 2.0, −1.6 ± 4.0, 17.1 ± 6.7, 70.2 ± 13.4, and ≥100 U/mL, respectively, for culture times of 0, 12, 24, 48, and 72 h. To further investigate the side-distribution of CA 15-3 antigens bound on the surface of the GaN MPs, we measured the fluorescence signal in the cross-sectional direction of 3 MPs, as illustrated in Fig. 7(c,d). The fluorescence signals were fully detected not only from the side-wall of the GaN MPs, but also from the bottom of the GaN template (Fig. 7(d)), showing that the fluorescence brightness was entirely uniform in a given area. In particular, the line profiles below the tips of the GaN MPs along the yellow line (A-A′) in Fig. 7(d) show almost the same fluorescence intensity without the background signal from the rear-positioned MPs owing to the sufficient distance between MPs. These results demonstrate that the GaN-based biosensors enabled the selective detection of the CA15-3 antigens from the cell suspension because the MCF-7 cells continually secreted various proteins, including the CA 15-3 antigen, during cell growth. Furthermore, by increasing the culture time to 72 h, the concentration of secreted CA 15-3 antigen exponentially increased to ≥100 U/mL. These fluorescence characteristics of the detected CA 15-3 antigens are correlated with the rapid increase of the MCF-7 population in the incubation period from 24 to 72 h^49^. Figure 8(a) shows overlapped fluorescence images of cultured MCF-7 cells as a function of culture time up to 72 h in an incubator. It indicates that the MCF-7 cells show enhanced cell differentiation with increasing incubation time. In addition, we also evaluated the population of MCF-7 cells dependent on the incubation time using a conventional hemocytometer (Hausser Scientific Co., USA). As shown in Fig. 8(b), the average number of cells was approximately 5.4 × 10^4^, 5.9 × 10^4^, 6.5 × 10^4^, 1.0 × 10^5^, 1.4 × 10^5^ and 1.6 × 10^5^ cells after an incubation of 0, 12, 24, 36, 48, and 72 h, respectively, revealing that the population of MCF-7 cells exponentially increased with increasing culture time up to 48 h. This growth behavior of the MCF-7 cells was similar to previous publication reported by Palizban and coworkers^49^. These results demonstrate that the GaN-based biosensors enabled the selective detection of the CA15-3 antigens from the cell suspension because the MCF-7 cells continually secreted various proteins, including the CA 15-3 antigen, during cell growth. Furthermore, by increasing the culture time to 72 h, the concentration of secreted CA 15-3 antigen exponentially increased to ≥100 U/mL. Based on preliminary experimental results showing the cell adhesion behavior of MCF-7 cells (Fig. 9), more detailed studies (i.e., direct and simultaneous determination of the concentration of tumor markers from blood samples of breast cancer patients) will be performed.

**Table 3:** CA 15-3 concentrations, calculated from the measured fluorescence intensity as a function of the culture time of MCF-7 cells (n = 3, 21 GaN MPs).

Culture duration of MCF-7 cell (h)	Measured fluorescence intensity	Calculated CA 15-3 concentration (U/mL)
0	17 ± 3	−3.6 ± 2.0
12	20 ± 6	−1.6 ± 4.0
24	48 ± 10	17.1 ± 6.7
48	127 ± 20	70.2 ± 13.4
72	211 ± 22	127 ± 13.4 (≥100)

## Conclusions

In summary, we successfully detected CA15-3 antigens secreted from MCF-7 cell lines using fluorescence-labeled GaN-MP-based biosensors. The high-quality GaN MPs were grown on the surface of hole-patterned GaN arrays with a MOCVD system using TMG and high-purity NH_3_ gas. The average diameter, length, and density of the MPs were determined to be ~1.2 μm, ~3.5 μm, and ~230,000 MPs/mm^2^, respectively, corresponding to a distance of ~3.2 μm between MPs. Through a series of chemical processes, CA15-3-Ab functionalized the STR-GaN MPs, which were mainly used to selectively detect CA15-3 antigens. According to the fluorescence images and intensities, which depend on the concentration of CA15-3 antigens bound on the functionalized GaN MPs, the fluorescence intensity showed a good linear correlation with the concentration of the CA15-3 antigen in the range from 0 to 80 U/mL. Based on this linear relationship between the fluorescence intensity and the concentration of CA15-3 antigens, the concentration of CA 15-3 antigens secreted from MCF-7 was determined to be 1.7 ± 3.4, 6.4 ± 4.0, 17.8 ± 3.4, 85.6 ± 12.1, and ≥100 U/mL, respectively, for MCF-7 cell suspensions containing ~1 × 10^3^, ~5 × 10^3^, ~1 × 10^4^, ~5 × 10^4^, and ~1 × 10^5^ cells/well. Furthermore, the concentration of the secreted CA 15-3 antigens was determined to be −3.6 ± 2.0, −1.6 ± 4.0, 17.1 ± 6.7, 70.2 ± 13.4, and ≥100 U/mL, respectively, for culture time 0, 12, 24, 48, and 72 h. This clearly demonstrates that the GaN-based biosensors enabled the selective detection of CA15-3 antigens from the proteins secreted during cell growth. The GaN MPs are therefore suitable for fluorescence sensing and for the measurement of the intensity of CA15-3 antigens secreted from the MCF-7 cell line because the concentration of CA15-3 for all breast cancer stages has been estimated to be in the range 20–120 U/mL^50^.

## Methods

### Fabrication of GaN MP Arrays

High-quality GaN films were grown on *c*-plane sapphire substrates with an MOCVD system using TMG as a Ga source and high-purity ammonia (NH_3_) as a N source. Prior to the GaN growth procedure, the substrates were thermally cleaned at 1080 °C for 5 min under H_2_ atmosphere and subsequently nitrided in NH_3_ ambient gas at thermal cleaning temperature for 5 min. Following this treatment, a GaN buffer layer with 20 nm thickness was deposited at 600 °C with a TMG/NH_3_ ratio of 15000. After the buffer-layer growth, the sample was heated up to 1040 °C in N_2_:H_2_:NH_3_ = 2:3:3 ambient conditions. Then, a GaN film with a thickness of approximately 2 μm was grown at a temperature of 1040 °C with TMG:NH_3_ = 15000:1. Subsequently, a thin SiO_2_ layer (thickness ~ 30 nm) was deposited on the surface of the grown GaN film using a plasma-enhanced chemical vapor deposition (PECVD) system. Photoresist hole patterns with size and period were constructed on the surface of the SiO_2_ film using a stepper lithography system, followed by anisotropic etching of the exposed SiO_2_ using an inductively coupled plasma-reactive ion etcher (ICP-RIE) and subsequent chemical dissolution of the photoresist in acetone. This process resulted in the formation of highly regular hole-patterned GaN arrays perforated into the SiO_2_ film. Finally, the GaN MPs were grown on the surface of the hole-patterned GaN arrays with the MOCVD system using TMG and high-purity NH_3_ gas at a temperature of 1000 °C and a pressure of 200 Torr, with H_2_ gas used as the carrier gas. One MOCVD cycle consisted of the following sequence: TMG supply (5 s, 15 sccm), TMG supply disruption (1 s), NH_3_ supply (10 s, 5 slm), and NH_3_ supply disruption (1 s). The resultant GaN MPs, with a height of 3.5 μm, were obtained after 300 cycles. After the removal of the remnant SiO_2_ film in a diluted hydrogen fluoride (10% HF) solution, the samples were rinsed with distilled water and dried with N_2_ gas.

### Surface Functionalization of GaN MPs

The GaN MPs grown on the *c*-plane sapphire substrates (25 mm × 25 mm) were first carefully cleaned with H_2_O_2_:H_2_SO_4_ (3:1) for 10 min to remove all of the organic materials and impurities from the surface. Then, the substrates were washed using a three-step cleaning process (acetone, isopropyl alcohol, and distilled water) and dried with nitrogen gas. The surface of the GaN MPs was treated with O_2_ plasma for 20 s to confer the hydroxyl groups on the surface and then modified with 1% (v/v) APTES in ethanol and at room temperature for 30 min using a 3D-rocker (100 rpm), resulting in amine group attachment onto the surface. After rinsing with an ethanol solution for 10 min, the GaN MPs were placed on a hot plate at 120 °C for 10 min and reacted with 12.5% (v/v) glutaraldehyde (GA, Sigma-Aldrich, USA) in distilled water for 4 h on a 3D-rocker. Subsequently, STR was immobilized to GA by incubating the GaN MPs with a 20 µg/mL STR solution in phosphate buffered saline (PBS) in an incubator (37 °C, 5% CO_2_) overnight. Finally, the STR-conjugated GaN MPs (STR-GaN MPs) were chemically functionalized with the CA15-3-Ab (×500 dilution) for 24 h in the incubator (4 °C, 5% CO_2_) and rinsed with PBS solution, resulting in CA15-3-Ab-functionalized STR-GaN MPs.

### Cell Sub-culture of MCF-7

The breast cancer cell line MCF-7 was purchased from the Korean Cell Line Bank (KCLB, Korea) and cultured in RPMI 1640 (phenol red-free) medium supplemented with 10% (v/v) fetal bovine serum and 1% (v/v) penicillin streptomycin at 37 °C in a 5% CO_2_ incubator. The MCF-7 cells were optically monitored daily to assess cell growth and population, and the culture medium was changed every two days. The cultured MCF-7 cells were passaged when they reached a confluence of approximately 80%.

The cell counts in a culture vessel were manually determined within an error of 10% using a conventional hemocytometer (Hausser Scientific Co., USA). Prior to loading the cell suspension onto the surface of the hemocytometer, the cultured cells were strained with Trypan Blue to distinguish dead from live cells. A series of counted and differently diluted cells in RPMI 1640 medium with a final volume of approximately 1 mL for each well was introduced into a 6-well culture plate with cell populations of approximately 1 × 10^3^, 1 × 10^4^, and 1 × 10^5^ cells/mL. In addition, a suspension containing ~1 × 10^5^ cells/mL was placed in the 6-well culture plate. After culturing the cells for up to 76 h at 37 °C in the 5% CO_2_ incubator, the culture medium extracted from the cell suspension was transferred into a 1 mL tube and then stored at 4 °C.

### Cell Staining

The cultured MCF-7 cells were first stained with DiI (Vybrant cell-labeling solution; membrane staining; 565 nm; Thermo Fisher Scientific, USA) and DAPI (4′,6-diamidino-2-phenylindole; nuclear staining; 470 nm; Thermo Fisher Scientific, USA) to investigate the cell growth behavior and population. The cells were rinsed at least twice with PBS (×1, Invitrogen, USA) to remove the culture medium (RPMI 1640), were stained with 1% DiI solution in PBS in an incubator at 37 °C for 40 min, and then washed with ×1 PBS solution. Subsequently, the DiI- and DAPI-stained MCF-7 cells were observed using a fluorescence microscope (EVOS^TM^, AMG, USA).
